# Comparative Metagenomic Analysis of Chicken Gut Microbial Community, Function, and Resistome to Evaluate Noninvasive and Cecal Sampling Resources

**DOI:** 10.3390/ani11061718

**Published:** 2021-06-09

**Authors:** Kelang Kang, Yan Hu, Shu Wu, Shourong Shi

**Affiliations:** 1 Poultry Institute, Chinese Academy of Agricultural Science, Yangzhou 225000, China; kangkelang@126.com (K.K.); huyan0128@126.com (Y.H.); wushu223759@163.com (S.W.); 2 Center of Effective Evaluation of Feed and Feed Additive (Poultry Institute) Ministry of Agriculture, Yangzhou 225000, China; 3 Jiangsu Co-Innovation Center for Prevention and Control of Important Animal Infectious Diseases and Zoonoses, Yangzhou 225000, China

**Keywords:** metagenome, bacterial community, resistome, sampling resources, chickens

## Abstract

**Simple Summary:**

Normally, researchers use feces or rectal swabs to characterize a gut microbiome, but because there is significant spatiotemporal variation across different intestinal segments there are marked differences in composition and function of microbiomes among various gut sites. Hence, a consensus has not been reached on the location for sampling for gut microbial metagenome sequencing. This study provides a comparative perspective on gut microbial function that takes into account different sampling resources when conducting a metagenomic sequence analysis, highlighting the differences in the choice of a gut microbiome sampling site, and investigating whether feces and rectal swab samples are efficient proxies for gut microbiome sampling.

**Abstract:**

When conducting metagenomic analysis on gut microbiomes, there is no general consensus concerning the mode of sampling: non-contact (feces), noninvasive (rectal swabs), or cecal. This study aimed to determine the feasibility and comparative merits and disadvantages of using fecal samples or rectal swabs as a proxy for the cecal microbiome. Using broiler as a model, gut microbiomes were obtained from cecal, cloacal, and fecal samples and were characterized according to an analysis of the microbial community, function, and resistome. Cecal samples had higher microbial diversity than feces, while the cecum and cloaca exhibited higher levels of microbial community structure similarity compared with fecal samples. Cecal microbiota possessed higher levels of DNA replicative viability than feces, while fecal microbiota were correlated with increased metabolic activity. When feces were excreted, the abundance of antibiotic resistance genes like *tet* and *ErmG* decreased, but some antibiotic genes became more prevalent, such as *fexA*, *tetL*, and *vatE*. Interestingly, *Lactobacillus* was a dominant bacterial genus in feces that led to differences in microbial community structure, metabolism, and resistome. In conclusion, fecal microbiota have limited potential as a proxy in chicken gut microbial community studies. Thus, feces should be used with caution for characterizing gut microbiomes by metagenomic analysis.

## 1. Introduction

The rapid development of culturomics and high-throughput sequencing (HTS) technologies have revealed that complex interactions exist between gut microbiota (the so-called “microbial organ” of a living body) and the host’s physiology, metabolism, homeostasis and immune system [1,2,3,4,5]. Regarding the optimal sampling location for gut microbial metagenome sequencing, a consensus has not yet been reached. In non-ruminants, the ceca are known to harbor a complex and dynamic microbial community [6], while the front and middle gut segments presented relatively lower abundance. In some studies, samples from rectal swabs were used to describe gut microbiota [7], while in other studies, sampling was limited to feces, which are convenient and easily accessible [8,9]. Collection is a non-intrusive and non-contact strategy that easily enables investigators to acquire samples in humans. Furthermore, as fecal sampling does not require animals to be sacrificed, continuity and temporal analysis for the dynamic tracking of the gut microbiome and monitoring of different life stages can be conducted for one particular animal. This is one possible explanation why feces sampling is often the preferred sampling method for gut microbiota [10]. By not harming or sacrificing any animals, investigators also minimize their distress, which ensures laboratory welfare in animal trials [11]. In addition, fecal samples have been reported to be the ideal specimen for detecting and studying antimicrobial-resistant genes, as many fecal bacteria including enterococci are exposed to antibiotic residues in this environment [12,13].

Despite the obvious advantages of fecal sampling and rectal swabs, a much-debated question is whether these microbiomes are representative of the gut microbiome. Previously, some researchers focused on aspects of diversity measurement for microbial communities using 16S rDNA sequence analysis, For instance, studies of C57BL/6J mice [14], humans [15,16], chickens [17], and other mammals [18,19] concluded that the microbiomes in feces, cecum, and mucus were distinct. Consequently, there is significant spatiotemporal variation across different intestinal segments [20] even within the same individual. However, other studies indicated that microbial diversity separation between cecal and fecal microbiomes was not as apparent in some experimental models such as obese animals [21]. Microbial diversity in the cecum and colorectum were similar, even among diverse chicken breeds [22]. Moreover, the gut environment consists mainly of anaerobic bacteria [23], and once feces are excreted, aerobes immediately start a new round of digestion and reproduction. Fecal microbiota were also reported to be qualitatively similar to cecal microbiota but quantitatively different.

Because of their varied microbial composition, gut sections have different microbiotic functions [24], but to date, these differences have received scant attention in the research literature, and this limits understanding of the interplay between the gut microbiome and cellular functions. Further, there is a need to understand spatiotemporal variations across different intestinal segments to learn more about the expression discrepancies in microbial genes, proteins, and other microbial products in the cecum and rectal chyme and feces. In summary, the microbial functions in the hindgut (cecum and rectum) of monogastric animals is poorly understood, and the efficacy of feces sampling for metagenomics and characterization of the gut microbiome needs further investigation. Elucidating microbial relationships between the hindgut (ceca and rectum) and feces could provide insight into gut microbial composition and functions and help researchers select sampling methods.

Herein, Arbor Acres (AA) broilers were used as a model to provide basic knowledge and a fresh perspective on microbial communities and functions in the chicken hindgut. The study aimed to compare hindgut microbial gene function by analyzing the total microbial gene, highlight the differences in sampling site location on the gut microbiome, and investigate whether fecal and rectal swab samples were efficacious as a proxy for the gut microbiome. To address these aims, total microbial genes were extracted, and metagenomic analyses were conducted to reveal the composition and function of the hindgut microbiome and fecal microbiome. The resulting data facilitated (1) a comparison of the number of microbial genes cataloged, (2) the identification and comparison of bacterial community composition, and (3) the prediction of the functional role of the microbiome in the chicken hindgut (Supplementary Figure S1a).

## 2. Materials and Methods

### 2.1. Chickens, Diets, and Sampling

AA broilers were obtained from Jing Hai Poultry Breeding, Co., Ltd. (Haimen City, China). This variety of chicken is widely reared for its superior growth and meat quality, and the mean age at slaughter is 42 days. Chickens (*n* = 45) were randomly divided into three repeats (with 15 broilers/repeats). All male broilers were hatched on the same day and reared in a poultry facility under standardized conditions during which birds were exposed to 24 h light. The chickens had free access to water and corn-soybean-based diets, based on the *Nutrient Requirements of Poultry: Ninth Revised Edition*, 1994 (NRC, 1994) and *Feeding Standard of Chicken* (NY/T 33-2004). The temperature of the poultry facility was controlled at 33 ± 0.5 °C on day 1 and then slowly decreased until it reached 26 °C on day 21, at which it was maintained.

At 42 days, to collect fecal samples, three chickens from each repeat were randomly selected and separately housed in a metabolic cage (Metabolic Cage, XiangShun Ltd., Guangzhou, China) for 3 days to adapt to the environment and relieve stress. Afterwards, fecal samples were collected into by sterile tweezers within 10 min of excretion; then, cloacal swab samples were collected by inserting sterile cotton swabs about 2 cm into the cloaca and turning slowly to absorb the chyme. Notably, the cloacal swab collected small bowel feces, rather than cecal contents. Finally, the chickens were slaughtered to collect cecal luminal contents. In each sampling resource, every 3 samples were mixed into a composite. Each resource had 3 composite samples containing 9 individuals, for a total of 27 from all three repeats. Samples were frozen using liquid nitrogen in 2 mL tubes, transported to the laboratory, and stored at −80 °C until subsequent analysis.

### 2.2. DNA Extraction and Library Preparation and Sequencing

Cetyltrimethylammonium bromide (CTAB) was used to extract DNA from the samples [25]. After they were ground in liquid nitrogen, Tris-HCL (pH 8.0), EDTA, NaCl and β-mercaptoethanol were added, and each sample was centrifuged for 10 min at 4000× *g*. The supernatant fraction was discarded, after which pre-warmed 2× CTAB and β-mercaptoethanol were added. The samples were then placed in a 65 °C water bath for 1 h. After one volume of chloroform/isoamyl alcohol (24:1) was added, the samples were incubated for 30 min and centrifuged for 10 min 3 times at 4000× *g*. The supernatant was collected and mixed with a 2× volume of anhydrous ethanol and kept at −20 °C for 40 min. After 10 min of centrifugation at 1000× *g*, the ethanol was carefully poured off and the sample was allowed to air dry for 15 min before finally being dissolved in ddH2O. Then DNA integrity was checked by agarose gel electrophoresis. The DNA obtained from each sample was diluted to 1 ng/μg with sterile water, and l μg was used as input material for the sequencing library preparations.

Sequencing libraries were generated using NEBNext^®^ Ultra™ DNA Library Prep Kit for Illumina (NEB, Ipswich, MA, USA). DNA was fragmented by sonication to the size of 350 bp, then the fragments were end-polished, a-tailed, and ligated using a full-length adaptor for Illumina sequencing with further PCR amplification. PCR products were purified (AMPure XP system) and libraries were analyzed for size distribution using an Agilent 2100 Bioanalyzer and quantified using a real-time PCR. Clustering of the index-coded samples was performed on the Illumina cBot Cluster Generation System; then, the library preparations were sequenced on an Illumina HiSeq platform and paired-end reads were generated.

### 2.3. Metagenome Assembly, Gene Prediction, and Abundance Analysis

After the sequencing, raw reads were cleaned by Readfq (version 8), and the data were subjected to a BLAST search against the host database using Bowtie (version 2.2.4) to filter the reads. The metagenome was assembled using a combination of single assembly and mixed assembly. For each sample, SOAPdenovo (version 2.04) was used to conduct single-sample assembly. Clean data from all samples were compared to each scaffold by using Bowtie software and acquiring unused paired-end reads. All samples were then combined, and SOAPdenovo and MEGAHIT (version 1.04-beta) were used for mixed assembly. Fragments shorter than 500 bp in the scaftigs generated from single or mixed assembly were filtered out for statistical analysis. MetaGeneMark (version 2.10) was then used to predict open reading frames (ORF) on scaftigs, and CD-HIT software (version 4.5.8) was employed to acquire the unique initial gene catalog. Clean data for each sample were mapped to the initial gene catalog using Bowtie to obtain the number of reads and statistical abundance of each gene in each sample.

### 2.4. Taxonomy Prediction and Gene Function Analysis

DIAMOND (version 0.9.9) was used to perform BLAST searches of unigenes to match sequences of bacteria, fungi, archaea, and viruses, which were all extracted from the NR database (version 2018-01-02) of the Nation Center for Biotechnology Information (NCBI). The parameter settings were blastp, −e 1e−5. A total of 794,741 genes were cataloged, of which 668,027 (84.06%) were annotated from the NR database and 14.28% were annotated as unclassified. As each sequence could have had multiple aligned results, the LCA algorithm (applied to system classification of MEGAN) was taken to ensure the accuracy of the species annotation information for sequences.

The unigenes were subjected to the BLAST analysis of functional databases including KEGG (version 2018-01-01), eggNOG (version 4.5), and CAZy (version 201801), by running DIAMOND with the parameter setting of blastp, taking the best hit with the standard e value ≤ 1 × 10^−5^. Resistance Gene Identifier (RGI) software was used to align the unigenes to the Comprehensive Antibiotic Resistance Database (CARD) with the parameter setting e value ≤ 1 × 10^−30^. The relative abundance of ARO was determined based on the results of the alignment.

### 2.5. Statistical Analysis

For diversity analysis, overall differences in microbial community structures were investigated using principal component analysis (PCA) and non-metric multidimensional scaling (NMDS) based on the abundance table of each taxonomic hierarchy using R ade4 and vegan package (version 2.15.3). Principal coordinate analysis (PCoA) was based on the Bray–Curtis distance value of abundance table for each taxonomic hierarchy. Metastats and LEfSe analysis were used to look for differences among the groups. A permutation test was used in Metastats analysis for each taxonomy to obtain the *p* value, then Benjamini and Hochberg’s False Discovery Rate was used to correct the *p* value and acquire the q value. The significance of differences among groups was checked by the Kruskal–Wallis test, and values of *p* < 0.05 were regarded as significant.

## 3. Results

### 3.1. Sequencing, Assembly, and Microbial Taxonomy

All processed samples were sequenced on an Illumina HiSeq, generating a total of 57,617.88 Mbp of raw data and an average of 6401.99 Mbp per sample. After cleaning raw reads with Readfq, an average of 6376.36 Mbp of clean data per sample remained, in which the percentage of non-host data was relatively low in cloaca, cecal chyme, and feces (Supplementary Table S1). After metagenome assembly by SOAPdenovo and prediction of open reading frames (ORFs) by MetaGeneMark, an average of 43.66% of reads (347,017 reads) were complete ORFs (1,452,753,985 bp scaftigs, 2,051,144 ORFs, and 794,741 gene catalogs). Samples from cloaca, cecum and fecal samples shared most of the 679,939 unigenes, and the number shared between the cloacal and cecal samples was much greater than that of the fecal samples alone (Figure S1b). Rarefaction curve analysis of all samples approached saturation, suggesting that mostly non-redundant genes of gut microbiota had been detected in different samples (Figure S1c). Correlation coefficients among samples indicated that those from an individual had relatively high similarity (Supplementary Figure S1d).

**Figure 1:**
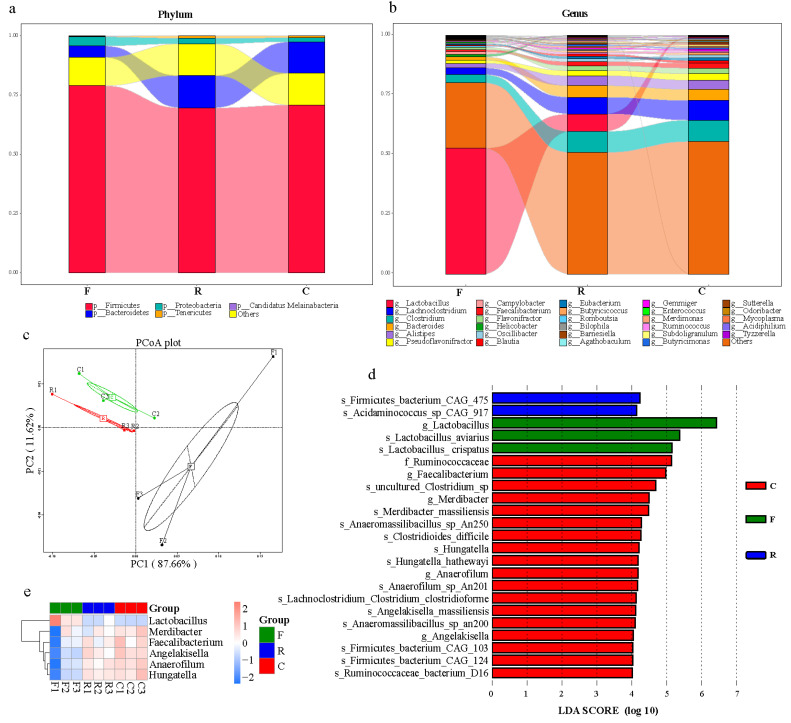
Microbial community structure from different sampling resources. (**a**) Relative abundance of dominant microbial phyla in different sampling resources. (**b**) Relative abundance of most abundant microbial taxa at the genus level in different sampling resources. (**c**) Principal coordinate analysis plot generated using abundance at different taxonomic levels based on Bray–Curtis dissimilarities. Each plot represents a sample. (**d**) LDA distribution histogram identified by the LEfSe algorithm. Only taxonomic biomarkers (LDA score > 4) are presented. (**e**) Clustering of taxonomic biomarkers based on LEfSe results. F, feces; C, cecal chyme; R, rectal chyme (inner cloacal chyme).

Homology searching was conducted with DIAMOND to characterize unigenes. A total of 85.72% were taxonomically classified at the kingdom level. Of these, between 90 and 98% were assigned to bacteria, followed by eukaryota, archaea, and viruses at a low abundance (less than 0.5%, Supplementary Table S2). For unigenes assigned as bacteria, samples from feces, cloaca, and cecum had similar dominant microorganism communities at the phylum level, including Firmicutes, Bacteroidetes, Proteobacteria, Tenericutes, and Candidatus Melainabacteria (Figure 1a). Despite the similarities in microbial community structures in all three samples, differences in microbial abundance were clearly visible. For instance, abundance of the phyla Tenericutes and Actinobacteria differed among the three resources (Kruskal–Wallis test, *p* = 0.039), with abundance in the feces significantly lower than in the cecum (Mann–Whitney test, *p* = 0.047, Supplementary Table S3).

At the genus level, *Lactobacillus* was the most abundant in feces within the phylum Firmicutes (Figure 1b), but its abundance differed in the three sampling resources (Kruskal–Wallis test, *p* = 0.027, Supplementary Table S4). *Lachnoclostridium*, *Clostridium*, and *Flavonifractor* were all less abundant in the feces (Mann–Whitney test, *p* < 0.05). The abundance of *Campylobacter* in the feces was higher than in the cecum, but *Faecalibacterium* showed the opposite trend (Mann–Whitney test, *p* = 0.047), while abundances of these two genera in the cloaca were in the middle. Microbial taxa at the genus level showed few differences between cloaca and cecum with the exception that *Lactobacillus* was more abundant in the cloaca than in the cecum (Mann–Whitney test, *p* = 0.049). Details of microbial diversity at the phylum, class, order, family, and genus levels are presented in Supplementary Figure S2. Furthermore, based on the Bray–Curtis distance, a dimensionality reduction analysis, the PCoA plots demonstrated that samples from feces clustered away from those of the cecum and cloaca in microbial structure (Figure 1c).

To differentiate the abundant bacterial taxa among cloacal and cecal chyme and feces, a linear discriminant analysis (LDA) effect size (LEfSe) was examined by the Wilcoxon rank test and gave an LDA score from microbial phylum to genus. A total of 23 distinct bacterial taxa were found in the three sampling resources. Members of the genus *Lactobacillus*, including *L. aviarius* and *L. crispatus*, were significantly abundant in feces, while *Firmicutes* bacterium CAG 475 and *Acidaminococcus* sp. CAG 917 were significantly abundant in the cloaca. Eighteen bacterial taxa were significantly abundant in the cecum (e.g., *Ruminococcaceae* and *Hungatella*; Figure 1d). The clustering of taxonomic biomarkers at the genus level revealed that *Lactobacillus* in feces was the highest. Furthermore, *Aecalibacterium*, *Anaerofilum*, *Merdibacter*, *Angelakisella*, and *Hungatella* in the feces were the lowest (Figure 1e, Supplementary Table S5).

### 3.2. Bacterial Functional Analysis

**Figure 2:**
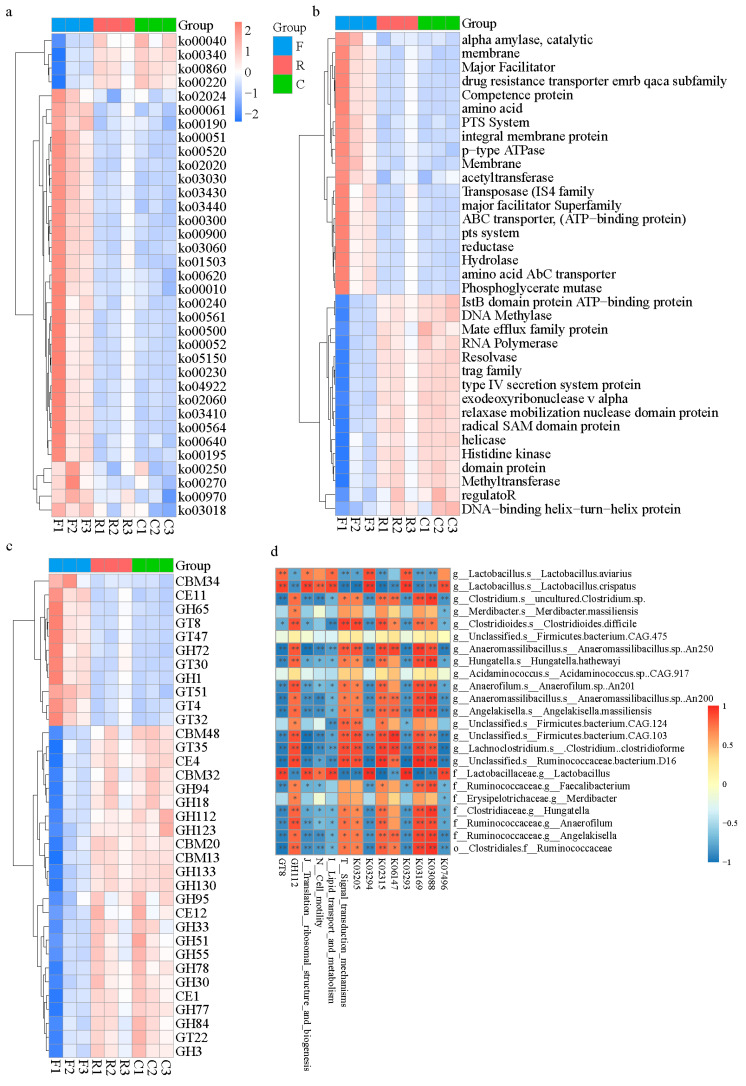
Microbial gene functions present in different sampling resources. (**a**) Microbial gene function-clustering-based heat map in KEGG database. (**b**) Microbial gene function-clustering-based heat map in eggNOG database. (**c**) Microbial gene function-clustering-based heat map in CAZy database. (**d**) Correlation between core microbiota and micro-function. The difference in relation to microbial functions was calculated by Spearman correlation test. Microbial functions are selected by LEfSe algorithm, blast all unigenes in KEGG, eggNOG and CAZy database. Red and blue titles indicate positive and negative correlations, respectively. Asterisks indicate significant correlations: *, *p* < 0.05; **, *p* < 0.01. F, feces; C, cecal chyme; R, rectal chyme (inner cloacal chyme).

After removing redundant sequences, 794,741 cataloged genes among the three samples were analyzed using the Kyoto Encyclopedia of Genes and Genomes (KEGG), the Evolutionary genealogy of genes: Non-supervised Orthologous Groups (eggNOG) and Carbohydrate-Active enzymes (CAZy) databases to reveal functional modules and pathways enriched in the microbiome (Supplementary Figure S3a–c). Overall, 63.84% of unigenes were assigned to KEGG pathways, 63.54% to the eggNOG database, and 3.21% to the CAZy database. Microbial gene function-clustering-based heat maps demonstrated that microbiota in the cloaca and cecum had similar gene functions (Figure 2a–c).

LEfSe analysis was employed to identify different microbial gene functions among the three databases; an LDA score > 3 reflected a significant difference among the three groups. In the KEGG database, three microbial genes in the feces, including putative transposase (K07496), amino acid transporter (K03293), and polyamine antiporter (K03294), and five genes in the cecum, including DNA topoisomerase III (K03169), DNA replication protein DnaC (K02315), type IV secretion system protein VirD4 (K03205), RNA polymerase sigma-70 factor (K03088), and ATP-binding cassette (K06147), were highly abundant based on an LDA score > 3 (Supplementary Figure S3d). In the eggNOG database, the microbial gene functions of the translation ribosomal structure and biogenesis, lipid transport and metabolism, and cell motility were all highly abundant in fecal samples compared with the others (LDA score > 3), while in the cecum, the only microbial genes with an LDA score > 3 were those involved in signal transduction mechanisms (Supplementary Figure S3e). For microbial genes that encoded enzymes involved in carbohydrate metabolism (CAZy database), the GT8 family in feces and the GH112 family in the cecum were highly abundant, which was different from the other samples (LDA score > 3), Supplementary Figure S3f). In general, the chyme in different parts of the chicken hindgut (in vivo or in vitro) significantly affected the divergence of microbial gene functions.

To explore the potential relevance of microbiota abundance and their function, correlation analyses were conducted. *Lactobacillus*, which is predominant in feces, showed significantly positive correlation with the putative transposase gene (K07496), amino acid transporter gene (K03293), and the polyamine antiporter gene (K03294), lipid transport and metabolism functions, cell motility, translation, ribosomal structure and biogenesis, and the GT18 family. In contrast, the correlation of *Firmicutes* bacterium CAG 475 and *Acidaminococcus* sp. CAG 917, which are predominant in the cloaca, with the above-mentioned functions was not significant. Furthermore, microbiota in the cecum presented numerous microbial metabolic activities. Microbiota in the feces were positively correlated with the GT8 family, while microbiota in the cecum showed a significant negative correlation. This trend was also observed in other pathways (Figure 2d). From the aspect of functional genes, microbiota in the cecum and cloaca of chickens differed from that in the feces.

### 3.3. Diversity and Abundance of Antibiotic Resistance

**Figure 3:**
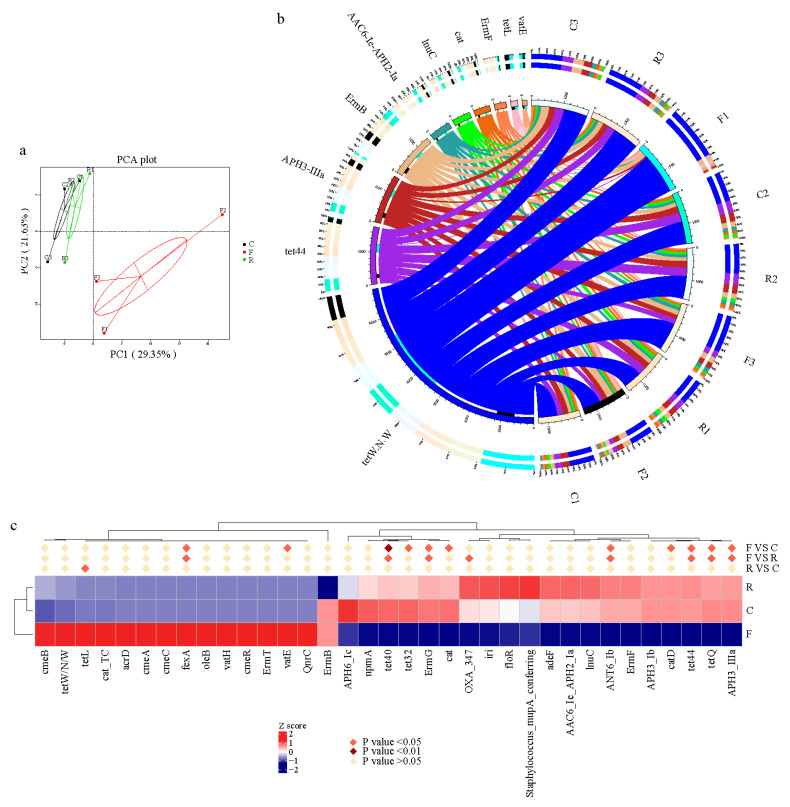
Variations of Antibiotic Resistance Ontology (ARO) subtypes in different sampling resources. (**a**) Principal component analysis of AROs. (**b**) Percentages of AROs in each sample: *tet*, tetracycline; *APH3*, phosphorylation of 2-deoxystreptamine aminoglycosides on the hydroxyl group at the 3′ position; *ErmB*, Erm 23S ribosomal RNA methyltransferase; *AAC6*, antibiotic inactivation of *APH2*; *LnuC*, lincosamide nucleotidyltransferase; *cat*, chloramphenicol acetyltransferase; *ErmF*, Erm 23S ribosomal RNA methyltransferase; *vatE*, streptogramin vat acetyltransferase. (**c**) Heatmap of variations of AROs based on the relative abundance of AROs. Statistically significant differences among different sampling resources are shown by different colors. F, feces; C, cecal chyme; R, rectal chyme (inner cloacal chyme).

Unigenes were annotated by the Comprehensive Antibiotic Resistance Database (CARD) to enhance understanding of the resistome in cloacal and cecal chyme and feces. Firstly, the relative percentages of Antibiotic Resistance Ontology (ARO) in each sample were classified (Supplementary Figure S4a and Table S6). The tetracycline-resistance gene family was inferred to have the highest relative abundance in each sample. There was shared similarity in resistome diversity between samples; cloacal, cecal chyme and feces have 205 AROs in common (Supplementary Figure S4b). The ARO variation presented by PCoA suggested that the feces resistome at the level of ARO differed from that of the cecum and cloaca (Figure 3a). For a more intuitive display, the top 10 ARO microbial taxa and their relative abundance in each sample were presented as a circular overview (Figure 3b). This revealed that the ARO of *tetW.N.W* showed maximum abundance, followed by *tet44*, *APH3-llla*, *ErmB*, *AAC6-le-APH2-1a*, *lnuC*, *cat*, *ErmF*, *tetL*, and *vatE*. The tetracycline resistance genes (*tet40*, *tet44*, and *tetQ*), *ErmG*, *ANT6* and *APH3* were significantly less abundant in feces (*p* < 0.05), whereas some ARO subtypes were significantly more abundant (*p* < 0.05), such as *fexA* (Figure 3c). Thus, it can be concluded that the abundance of the tetracycline resistance gene *fexA* and other antibiotic resistance genes changed when cecal chyme was excreted.

## 4. Discussion

Next-generation sequencing (NGS) technology is a revolutionary change over traditional sequencing [26], and has widened our view of the microbiome. However, research on the gut microbiome to reveal its structure and function is largely dependent on the sampling site [17], as spatial localization (in vitro or in vivo) has a substantial effect on microbial metabolism and growth. It has been observed that microbial population heterogeneity is prevalent in cecal and fecal microbiota by conducting 16S rDNA sequencing [14,17,27], but microbial gene function and resistome heterogeneity have not been comprehensively investigated, which metagenomic sequencing are needed.

For accessibility, sampling feces is more convenient than other sampling resources because it enables researchers to obtain gut microbial samples without killing and touching animals. Moreover, more time and greater effort and cost are required to collect cecal chyme than feces [28]. In this study, NGS was used to characterize various facets of the chicken gut microbiome obtained from three sampling methods (feces, rectal swabs, and cecal chyme). Microbial composition, function, and resistome were examined to reveal the advantages and disadvantages of sampling different gut segments and using different methods.

The dominant phyla in the hindgut and feces were Firmicutes, Bacteroidetes, and Proteobacteria, which was consistent with previous studies on broilers [29,30,31,32]. A similar relative abundance of Firmicutes was observed between our study (>69% in different intestinal compartments) and a previous study on broilers reported by [33], 76.2%). The relative abundance of Bacteroidetes in the cecum and cloaca was much higher than that in the feces. Firmicutes and Bacteroidetes are predominant in many mammalian gut catalogs [34,35]. Members of these two phyla are capable of producing short-chain fatty acids (SCFAs) in the colon [36] and play a vital role in regulating host energy metabolism. At the genus level, *Lactobacillus* (Firmicutes), *Lachnoclostridium* (Firmicutes), *Clostridium* (Firmicutes), and *Bacteroides* (Bacteroidetes) were the four dominant genera in the cecum, cloaca, and feces. *Lactobacillus* was the most abundant genus in cloacal (7.30%) and fecal (52.70%) samples, but was not very abundant in cecal samples (0.35%). Most microbial taxa in the cecum and cloaca could be identified in the feces; hence, samples from the three different parts of the chicken gut had the same microbial community membership. However, substantial variations in the abundance of the genera *Lactobacillus*, *Lachnoclostridium*, *Clostridium*, and *Bacteroides* were observed at all three sites. In summary, there were differences in microbial diversity among the three sources with samples from the cecum and cloaca exhibiting a much higher similarity to each other than to fecal samples. This is likely due to environmental heterogeneity between the different intestinal compartments. Thus, even when a single animal is used, there may be microbial variation because the different sampling sites in the animal are subject to environmental heterogeneity, which may be derived from pH [37], oxygen exposure [38], or substrate concentrations [19].

Earlier studies based on 16S rRNA gene sequencing and using operational taxonomic units (OTUs) to compare microbial diversity, failed to reach agreement on whether to use using feces or rectal swabs as a proxy for animal gut microbiota. In contrast to our study, it was reported that fecal and cecal samples shared most of the OTUs [17]. This was attributed to the foregut microbiota being the primary determinant of fecal microbiota composition, with chyme eventually becoming feces [39]. However, Wen et al. (2019) found that the abundances of microbiota in different parts of the chicken gut, including the small intestine, ileum, cecum, and feces, were totally different at the phylum and genus levels [29]. In the present study, cecal sampling was found to be beneficial for focusing on bacterial composition, because the cecum, representative of the internal gut environment, showed higher microbial diversity than did the feces. From the sequence analysis above, investigations into gut microbial community structure should sample cecum chyme rather than feces to obtain metagenome sequences. Furthermore, even rectal swabs would be preferable, as the gut microbiome from rectal swabs was similar to the cecal microbiome.

Cecal microbiota play an important role in digesting dietary crude fiber, which affects nutrient digestion and absorption in chickens [40,41]. Consequently, the cecal microbiome was widely investigated [17]. In our study, cecal chyme had 18 specific microbial taxa (LDA > 4), much more than the cloacal and fecal samples, and most of these taxa were positively correlated with genes encoding DNA topoisomerase III, DNA replication protein DnaC, type IV secretion system protein VirD4, RNA polymerase sigma-70 factor, ATP-binding cassette, and GH112 family members. During cell replication, DNA topoisomerase is activated a number of times to regulate DNA supercoiling [42,43]. The type IV secretion system protein VirD4 mediates the transport of effector proteins or DNA through the cell membrane of Gram-negative bacteria, so is referred to as a “Chaperone protein” [44,45], and it facilitates communication channels between gut microbiota. RNA polymerase sigma-70 factor binds to the core polymerase, allowing it to recognize a specific DNA sequence as a promoter. Taken together, it can be inferred that sampling cecal chyme for sequencing is more suitable for analysis of gut microbial functions due to the higher level of gene replication activity in the cecal chyme samples.

Regarding the fecal samples, those in present study had a high abundance of *Lactobacillus*, which has a long history as an exogenous probiotic [46]. Feces are also associated with other microbial activities, such as amino acid transporter, related to the first step in synthesizing bacterial protein, and it is responsible for the interaction with gut microbiota [47]. The putative transposase is an activator of microbial mobile elements, and it targets horizontal gene transfer [48]. Microbial-derived polyamines have many biological functions related to host health and disease [49]. *Lactobacillus* demonstrated a significant positive correlation with these microbial functions, indicating that chicken feces still possesses rich nutrients for microbial fermentation. *Lactobacillus* was also positively correlated to GT8 family members, which are involved in lipopolysaccharide biosynthesis [50]. When cecal chyme was excreted and gradually became feces, the relative abundance of *Lactobacillus* increased as did other functional activity such as GT8, lipid transport and metabolism. Hence, when conducting research to reveal gut microbial function, cecal samples are needed because they present high microbial diversity and microbial bioactivity.

Previous studies of antibiotic resistance using traditional culture-based methods raised awareness of a diverse range of emerging antibiotic resistance genes (ARGs) [51,52], in broilers under different antibiotic treatments, among different farms, and in different breeds [22]. In the current study, NGS was adopted to compare the presence and relative abundance of ARGs in the feces, cloaca, and cecum of broilers, to find the most suitable sampling method and gut-sampling segment. It was concluded that ARG diversity in the cecum and the cloaca was similar, while that in feces was not. The *tetW.N.W* and *tet44* ARGs had the highest and second-highest abundance, respectively, of all those detected. In recent decades, tetracycline has been widely incorporated into animal feed to minimize diseases and promote growth rates [53]. Although the use of antibiotics as growth promoters was banned more than 10 years ago, ARGs had already been deposited on microbial genomes [54]. Hence, the observation that the chicken gut microbiome has a broad ranging resistome (more than 200 types of ARO) is not surprising. The aminoglycoside phosphotransferase APH (3′)-IIIa inactivates amikacin, kanamycin, and neomycin [55], and as the relative abundance of APH (3′)-IIIa in the feces decreased, it suggested that the abundance of bacteria that carried these kinds of ARGs decreased as the chyme was turned into feces and excreted. The florfenicol resistance gene, *fexA*, is carried on a plasmid and was first described in *Staphylococcus* [56]. In the present study, the relative abundance of *fexA* was significantly higher in the feces than in the cecum and cloaca, while the relative abundance of many tetracycline resistance genes (e.g., *tet40*, *tet44*, and *tetQ*) decreased significantly in the feces compared with the cecum and cloaca. These findings supplemented our understanding of ARGs carried by chicken gut microbiota and supported the hypothesis that the composition of ARGs at these sites would be different because of the distinct microbial community structure and diversity of both feces and the internal environment of the chicken gut. In summary, when studying ARGs, sampling strategies need to be tailored to different subtypes of target ARGs because of the many decreases and increases in ARG subtypes in the feces, cecum, and rectum.

Taking all of the above into account, cecal chyme is recommended for evaluating microbial diversity and species richness, while rectal swabs and fecal samples are more applicable for continuous individual monitoring. Additionally, for studies focused exclusively on the resistome, which may cause serious pollution to the environment, sampling feces is the optimal choice.

## 5. Conclusions

Feces have a higher abundance of *Lactobacillus* and higher metabolic activity than the cecal and cloacal samples, whereas cecal samples have the highest replication activity. Using fecal samples as a proxy for studying the gut microbial community and function of broilers showed limited efficacy although sampling feces has certain advantages for convenience and animal welfare. However, they are more suitable for detecting antibiotic resistance than rectal and cecal samples. In conclusion, using feces to represent the hindgut microbiota is of limited efficacy, and researchers should consider employing different sampling strategies for different purposes.

## Data Availability

These sequence data have been submitted to the Biotechnology Information (NCBI) Sequence Read Archive databases under accession number PRJNA658643.

## Supplementary Materials

The following are available online at https://www.mdpi.com/article/10.3390/ani11061718/s1, Figure S1: (**a**) Sampling strategy when conducting metagenomic analysis of gut microbiome. (**b**) Venn diagram of the total number of genes in each group. (**c**) Rarefaction curves of detected genes from the whole samples. (**d**) Correlation between each sample, the darker the color is and the flatter the ellipse is, the greater the absolute value of the correlation coefficient between samples, Figure S2: Distribution of the most abundant taxa of cecal microbiota in different samples. (**a**) phylum; (**b**) class; (**c**) order; (**d**) family; (**e**) genus, Figure S3: Microbial gene function annotation. (**a**) KEGG pathway annotation. (**b**) eggNOG pathway annotation. (**c**) CAZy pathway annotation. (**d**–**f**) Microbial gene function annotation based on LDA score, Figure S4: Variations of ARO subtypes in different part of sampling. (**a**) Relative abundance of dominant ARO subtypes in each samples. (**b**) Venn diagram of ARO shared between feces, rectum and cecum. F, feces; C, cecal chyme; R, rectal chyme (inner cloacal chyme), Table S1: quality control result of each samples, Table S2: percentage of contigs assigned in different Kindom(%), Table S3: relative abundance (%) of the predominant microbiota at the phylum level in different group, Table S4: relative abundance (%) of the predominant microbiota (top 10) at the genus level in different part of sampling, Table S5: relative abundance (%) of microorganisms (LDA > 4) clustered in genus level, Table S6: relative aboudance (%) of Antibiotic Resistance Gene in each sample.
