# Supplementary figures and images for: Comparative Metagenomic Analysis of Chicken Gut Microbial Community, Function, and Resistome to Evaluate Noninvasive and Cecal Sampling Resources
Source: Animals (Basel). 2021 Jun 9;11(6):1718. doi: 10.3390/ani11061718 (PMC8228302; doi:10.3390/ani11061718)

## KEGG pathway annotation

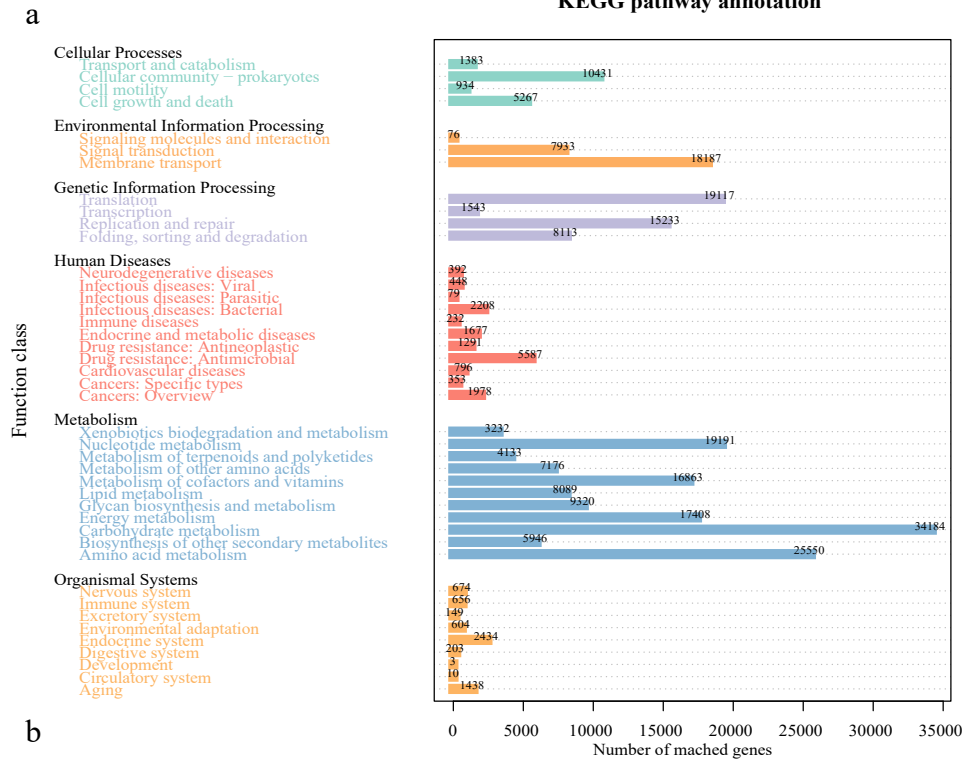

**d**

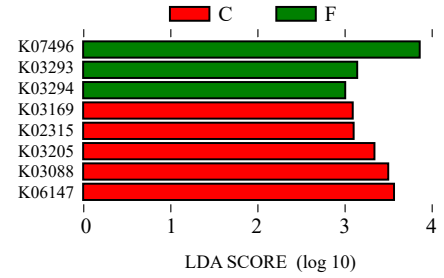

**b**

## eggNOG pathway annotation

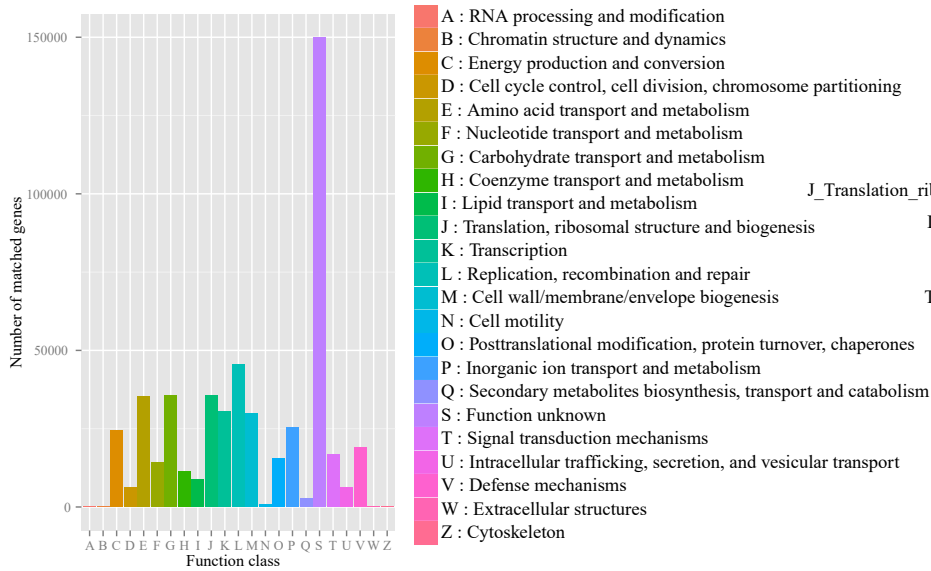

**e**

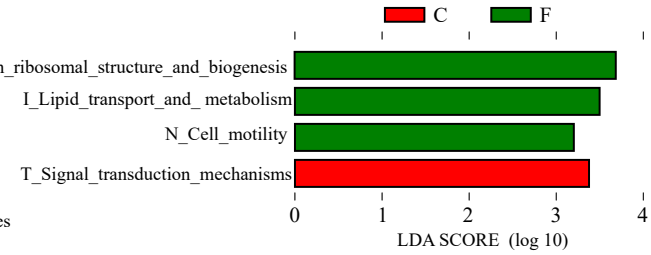

**c**

## CAZy pathway annotation

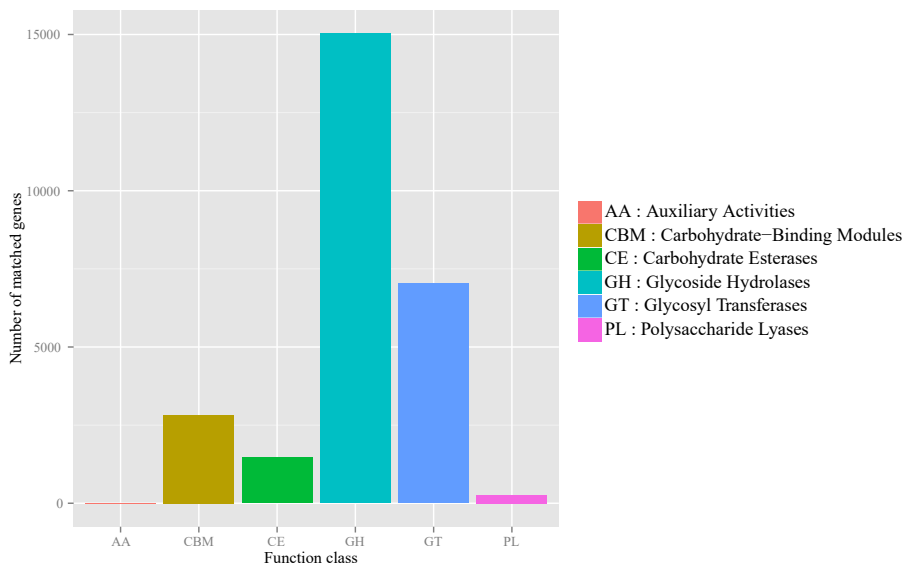

**f**

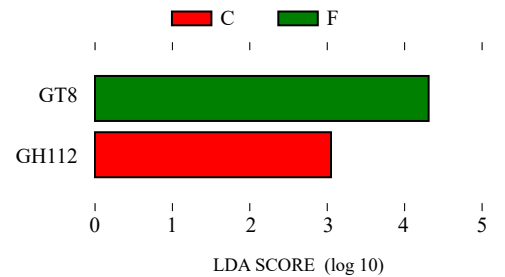

Supplement: Supplementary file 1 [file animals-11-01718-s001.zip › FigureS3.pdf]

a

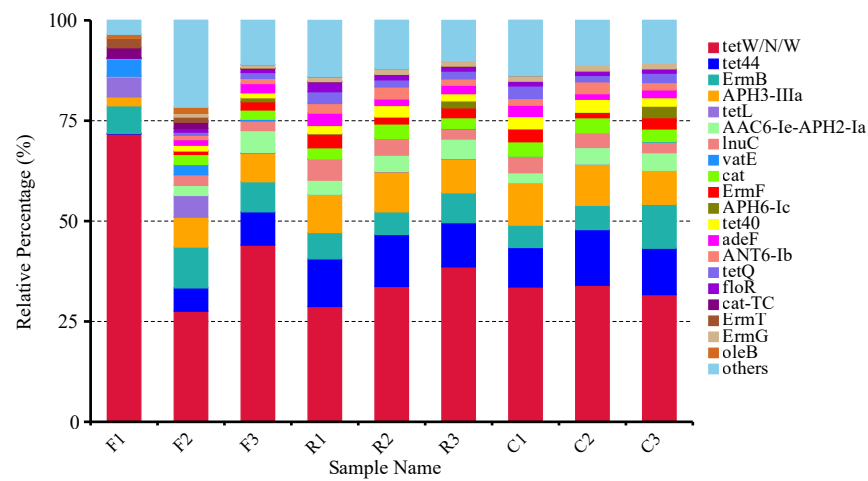

b

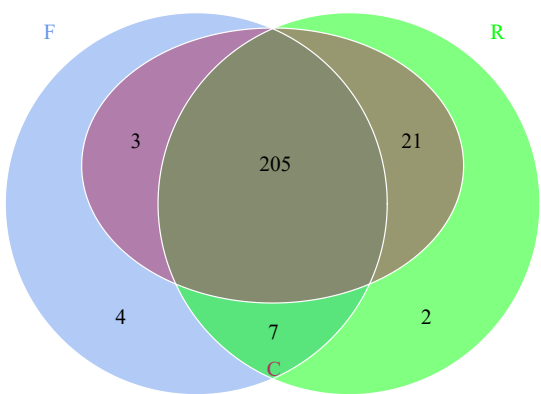

Supplement: Supplementary file 1 [file animals-11-01718-s001.zip › FigureS4.pdf]

a

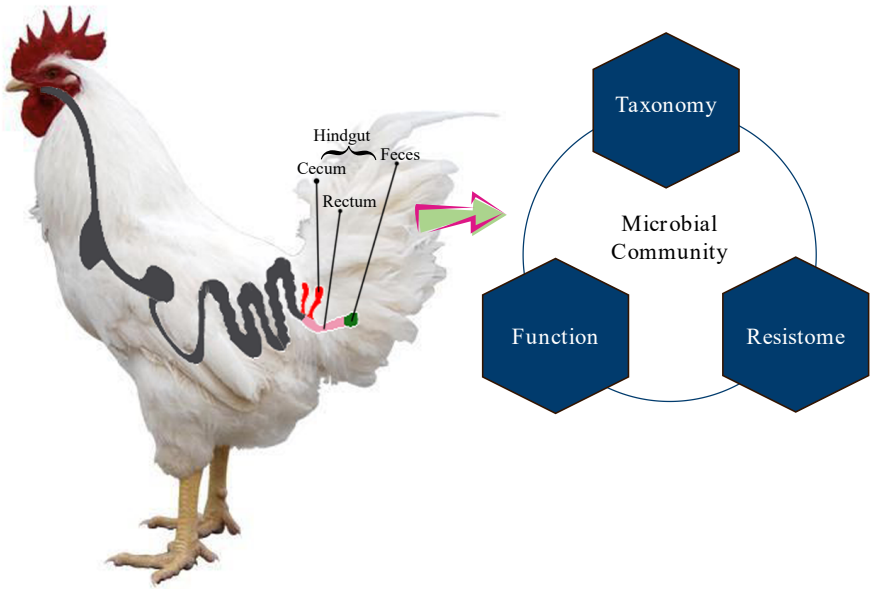

b

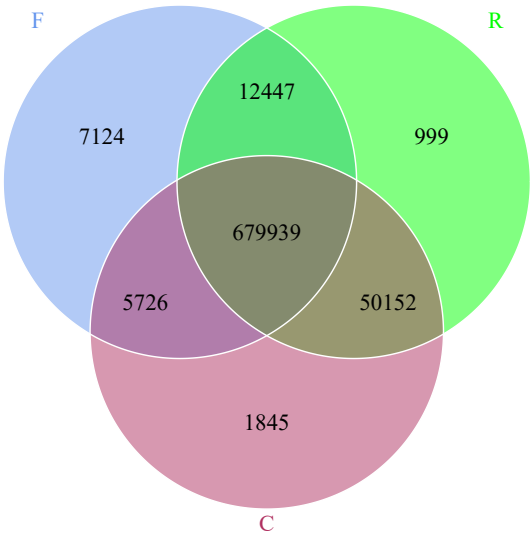

c

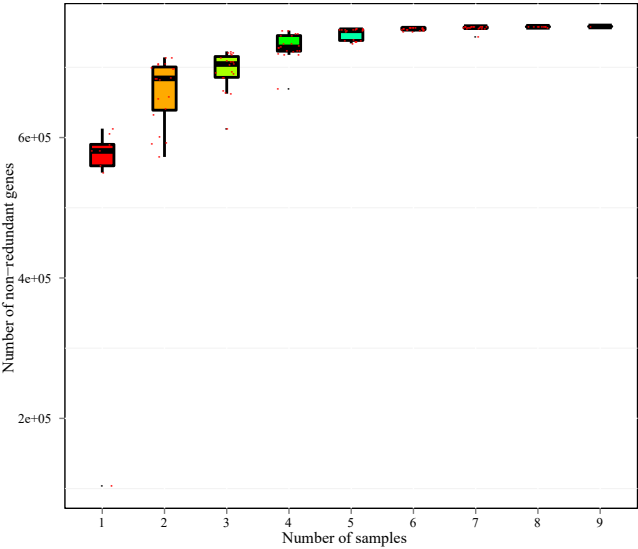

d

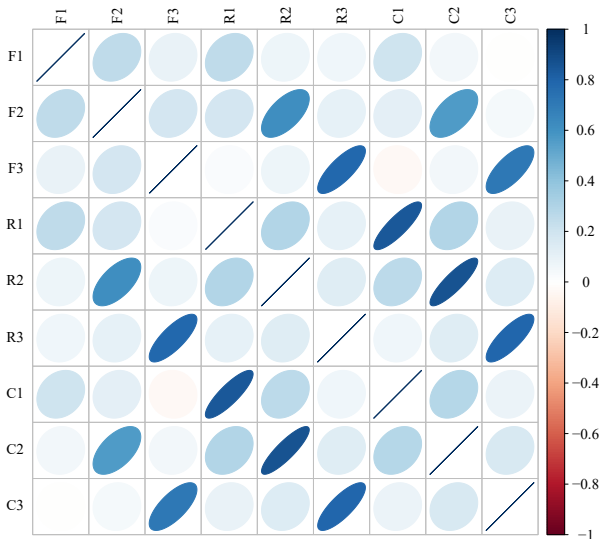

Supplement: Supplementary file 1 [file animals-11-01718-s001.zip › figureS1.pdf]

a

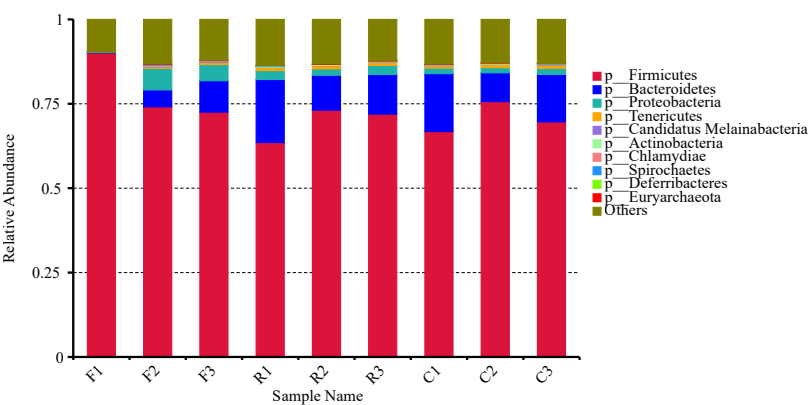

b

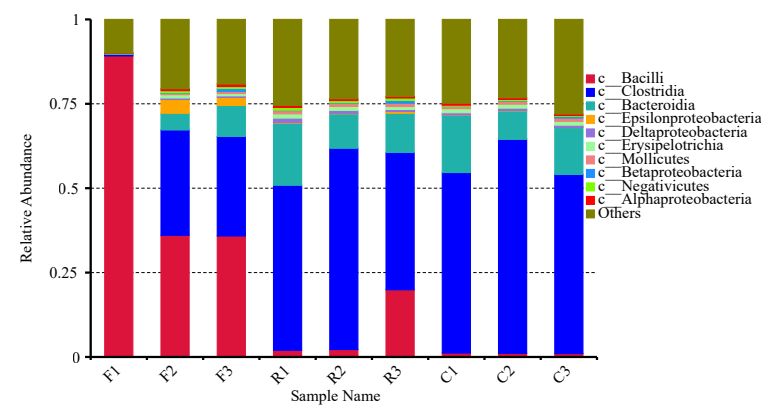

c

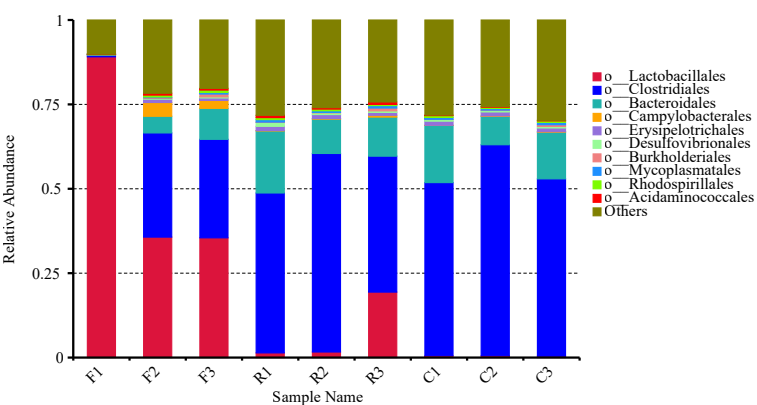

d

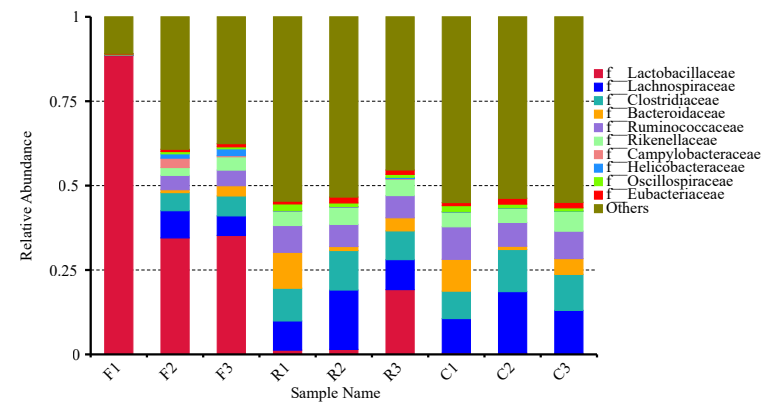

e

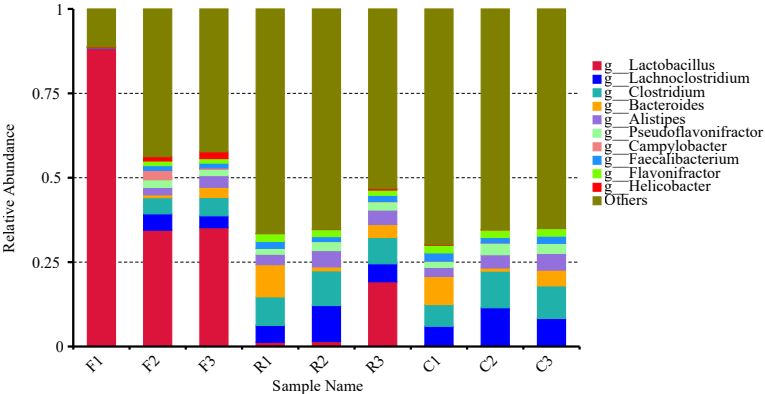

Supplement: Supplementary file 1 [file animals-11-01718-s001.zip › FigureS2.pdf]
